# General Phenotype of NADase Induction by CLI Treatment in *Streptococcus pyogenes*

**DOI:** 10.1155/2022/4767765

**Published:** 2022-10-26

**Authors:** Ichiro Tatsuno, Masanori Isaka, Tadao Hasegawa

**Affiliations:** Department of Bacteriology, Nagoya City University Graduate School of Medical Sciences, 1 Kawasumi Mizuho-cho Mizuho-ku, Nagoya 467-8601, Japan

## Abstract

The administration of high-dose clindamycin (CLI) along with penicillin is recommended for the treatment of streptococcal toxic-shock syndrome (STSS). However, we previously reported that a “subinhibitory dose” of CLI induced the expression of the NAD-glycohydrolase (NADase) exotoxin in an *emm*1-type *Streptococcus pyogenes* 1529 strain isolated from an STSS patient. In this study, we examine NADase induction by CLI treatment using an extracellular NADase activity assay instead of the previous two-dimensional gel electrophoresis assay. The examination revealed that CLI administration can induce NADase expression in a dose-dependent manner. We analyzed 23 CLI-susceptible strains (5 *emm*1 strains, 6 *emm*3 strains, 3 *emm*4 strains, 1 *emm*6 strain, 3 *emm*12 strains, 1 *emm*28 strain, and 4 *emm*89 strains), and 19 of the 23 strains showed similar NADase induction phenotypes to that shown in strain 1529. These results indicate that NADase induction by CLI treatment is not restricted to specific strains and it could be a standard phenotype among CLI-susceptible *S. pyogenes* strains. We also analyzed four CLI-resistant strains. All four strains showed increased extracellular NADase activities at high concentrations of CLI that did not inhibit bacterial growth. These results indicated that the subinhibitory dose of CLI was not the critical factor for NADase induction.

## 1. Introduction

*Streptococcus pyogenes* is a Gram-positive bacterium that infects the upper respiratory tract, including the tonsils and pharynx, and it is responsible for postinfection diseases, such as rheumatic fever and glomerulonephritis. *S. pyogenes* also causes severe invasive diseases, including necrotizing fasciitis and streptococcal toxic-shock syndrome (STSS) [1–5].

The administration of high-dose clindamycin (CLI) along with penicillin is recommended for the treatment of STSS. However, in our previous study, the levels of two exoproteins, streptolysin O (Slo) and NAD (+)-glycohydrolase (Nga or NADase), were increased by CLI treatment (named “CLI-dependent Slo/NADase induction”) in three of the five STSS strains analyzed by two-dimensional gel electrophoresis [6]. The NADase, the expression of which is negatively regulated by the CovRS two-component regulatory system [7, 8], is an important virulence factor for STSS [9]. We also have studied NADase [10–12]. In this study, we examined the CLI-dependent NADase induction using a more quantitative extracellular NADase activity assay than the previous two-dimensional gel electrophoresis assay. Consequently, the examination provided new insights into CLI-dependent NADase induction.

## 2. Materials and Methods

### 2.1. Strains

We collected *S. pyogenes* strains isolated from Nagoya City University Hospital and some hospitals mainly in the Aichi Prefecture of Japan. *S. pyogenes* SSI-1, the database reference strain, was provided courtesy of Dr. Kawabata [13]. In accordance with the guidelines of the Clinical and Laboratory Standards Institute, strains with a minimal inhibitory concentration for CLI exceeding 1 *μ*g/ml were defined as resistant to CLI (https://www.clsi.org/).

### 2.2. Culture Conditions

Bacteria were cultured in brain heart infusion broth (Eiken Chemical, Tokyo, Japan) supplemented with 0.3% yeast extract (Becton Dickinson, Sparks, MD, USA) (BHI-YE) at 37°C without agitation, as described previously [14].

### 2.3. Determination of emm Type

Typing of the M protein-coding gene (*emm*) of streptococcal isolates was performed according to the methods by CDC *Streptococcus Laboratory* (https://www.cdc.gov/streplab/protocol-emm-type.html) as described previously [14].

### 2.4. Quantification of NADase Activity in Bacterial Supernatants

A 40 *μ*L sample of the overnight culture was added to 4 ml of fresh BHI-YE broth containing CLI at various concentrations, and the samples were cultured for 18 h. After 18 h, the optical density at 660 nm (OD_660_) was measured. The NADase activity of the culture supernatants was determined using the method of Stevens et al. [15] as described previously [10].

## 3. Results

### 3.1. Effects of CLI on Extracellular NADase Activity in emm1 Strains

**Figure 1:**
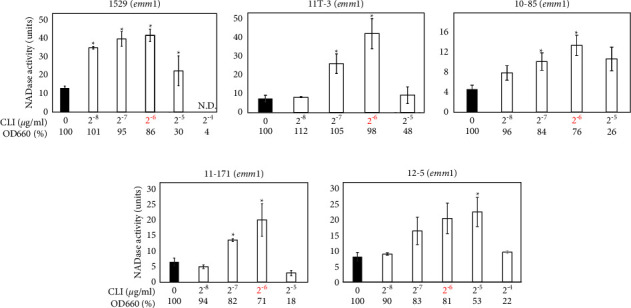
Extracellular NADase activity in *emm*1-type strains cultured with clindamycin (CLI). One unit of NADase activity was defined as the amount (*μ*g) of *β*-NAD cleaved per hour per *μ*L of culture supernatant, as described previously [10]. The OD_660_ of overnight culture with CLI (2^−8^ to 2^−4^ *μ*g/ml) is shown by a percentage (%) of that without CLI (0 *μ*g/ml). The maximum CLI concentration that did not suppress the bacterial growth to less than 50% of that without CLI is shown in red, which was 2^−6^ *μ*g/ml in strains 1529, 11T-3, 10–85, and 11–171, and 2^−5^ *μ*g/ml in strain 12–5. At least three independent experiments were performed. Error bars indicate the standard errors of the means. Significant increases in extracellular NADase activity by CLI treatment when compared with that without CLI treatment, shown by the black bar, are marked with asterisks (^*∗*^*p* < 0.05 using Student's *t*-test).

In the previous study [6], a subinhibitory dose of CLI induced the expression of NADase in an *emm*1 *Streptococcus pyogenes* 1529 strain isolated from an STSS patient. The subinhibitory dose was defined as the maximum antibiotic concentration that did not suppress bacterial growth in the previous study [6]. However, it is unknown whether the subinhibitory dose is the best concentration to induce the expression of NADase because other concentrations were not tested. Therefore, we examined the previous study using various CLI concentrations (Figure 1 and Table 1). The bacterial growth was measured using the OD_660_ (see Materials and Methods for detail), which was the method used in the previous study [6]. When strain 1529 was cultured for 18 h without CLI (0 *μ*g/ml), the average OD_660_ was 1.03. When cultured with 2^−8^, 2^−7^, 2^−6^, and 2^−5^ *μ*g/ml CLI, the average OD_660_ values were 1.05, 0.98, 0.88, and 0.31, respectively. The OD_660_ values listed were 101%, 95%, 86%, and 30% of the OD_660_ without CLI, respectively (Figure 1). The subinhibitory dose was newly defined as the maximum CLI concentration that did not suppress the bacterial growth to less than 50% of that without CLI. The new subinhibitory dose was 2^−6^ (≈1.6 × 10^−2^) *μ*g/ml in strain 1529 (shown in red and black in Figure 1 and Table 1, respectively). The extracellular NADase activities were increased from 2^−8^ to 2^−6^ *μ*g/ml CLI in a dose-dependent manner, and the NADase activity reached the maximum in the 2^−6^ *μ*g/ml CLI, the new subinhibitory dose (Figure 1).

Strain 1529 was the only *emm*1-type strain analyzed in the previous study [6]. In the current study, we analyzed an additional four *emm*1 strains (11T-3, 10–85, 11–171, and 12–5) isolated from STSS or non-STSS patients, and these strains showed similar NADase induction phenotypes to those shown in strain 1529 (Figure 1 and Table 1).

### 3.2. Effects of CLI on Extracellular NADase Activity in emm3, emm4, and emm12 Strains

**Figure 2:**
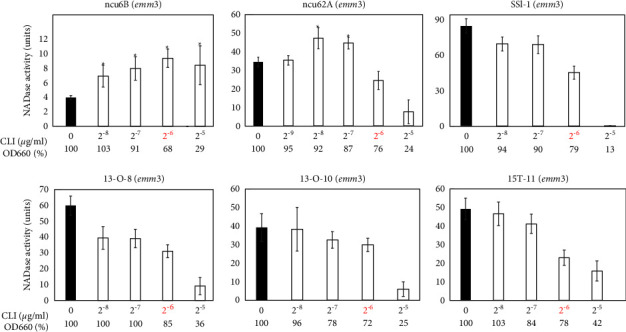
Extracellular NADase activity in *S. pyogenes emm*3-type strains cultured with CLI. NADase activity was determined based on the criteria described in Figure 1. The OD_660_ of overnight culture with CLI (2^−8^ to 2^−5^ *μ*g/ml) is shown by a percentage (%) of that without CLI (0 *μ*g/ml). The maximum CLI concentration that did not suppress the bacterial growth to less than 50% of that without CLI is shown in red, which was 2^−6^ *μ*g/ml in all the strains. At least three independent experiments were performed. Error bars indicate the standard errors of the means. Significant increases in extracellular NADase activity by CLI treatment when compared with that without CLI treatment, which is shown by the black bar, are marked with asterisks (^*∗*^*p* < 0.05 using Student's *t*-test).

**Figure 3:**
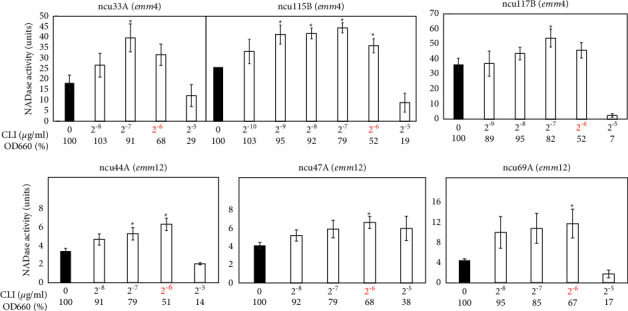
Extracellular NADase activity in *emm*4-or *emm*12-type strains cultured with CLI. NADase activity was determined based on the criteria described in Figure 1. The OD_660_ of overnight culture with CLI (2^−10^ to 2^−5^ *μ*g/ml) is shown by a percentage (%) of that without CLI (0 *μ*g/ml). The maximum CLI concentration that did not suppress the bacterial growth to less than 50% of that without CLI is shown in red, which was 2^−6^ *μ*g/ml in all three strains. At least three independent experiments were performed. Error bars indicate the standard errors of the means. Significant increases in extracellular NADase activity by CLI treatment when compared with that without CLI treatment, which is shown by the black bar, are marked with asterisks (^*∗*^*p* < 0.05 using Student's *t*-test).

In the previous study [6], not only strain 1529 but also four additional strains (1268-*emm*3, 1266*-emm*4, 1547-*emm*5, and GG01*-emm*12) were analyzed. The 1547-*emm*5 and GG01*-emm*12 strains showed CLI-dependent NADase induction, whereas strains 1268-*emm*3 and 1266*-emm*4 did not. In order to investigate how the difference between the former two and the latter two strains in the NADase induction phenotypes is related to their *emm*-genotypes, an additional twelve strains (six *emm*3, three *emm*4, and three *emm*12) were analyzed, although we could not collect additional *emm*5 strains. Among the six *emm*3 strains, two strains (ncu6B and ncu62 A) showed CLI-dependent NADase induction, although the other four strains (SSI-1, 13-O-8, 13-O-10, and 15-T-11) did not (Figure 2 and Table 1). All *emm*4 and *emm*12 strains showed CLI-dependent NADase induction (Figure 3 and Table 1). These results indicated that the *emm*3-and *emm*4-genotypes were not attributable to “no NADase induction” phenotypes that were previously reported for the 1268-*emm*3 and 1266*-emm*4 strains [6].

### 3.3. Effects of CLI on Extracellular NADase Activity in the emm6, emm28, and emm89 Strains

**Figure 4:**
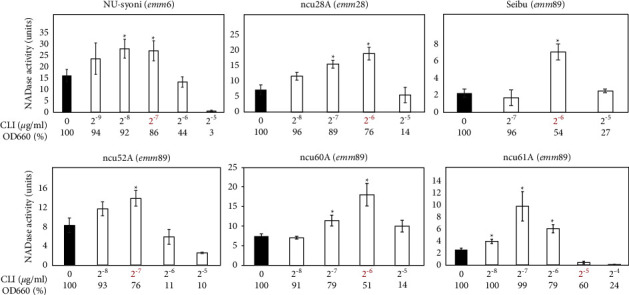
Extracellular NADase activity in *emm*6, *emm*28, or *emm*89 strains cultured with CLI. NADase activity was determined based on the criteria described in Figure 1. The OD_660_ of overnight culture with CLI (2^−9^ to 2^−4^ *μ*g/ml) is shown by a percentage (%) of that without CLI (0 *μ*g/ml). The maximum CLI concentration that did not suppress the bacterial growth to less than 50% of that without CLI is shown in red, which was 2^−7^ *μ*g/ml in strain NU-syoni, 2^−6^ *μ*g/ml in strains ncu28A and Seibu, 2^−7^ *μ*g/ml in strain ncu52 A, 2^−6^ *μ*g/ml in strain ncu60 A, 2^−5^ *μ*g/ml in strain ncu61A, respectively. At least three independent experiments were performed. Error bars indicate the standard errors of the means. Significant increases in extracellular NADase activity by CLI treatment when compared with that without CLI treatment, which is shown by the black bar, are marked with asterisks (^*∗*^*p* < 0.05 using Student's *t*-test).

We analyzed one *emm*6, one *emm*28, and four *emm*89 strains. All six strains showed the CLI-dependent NADase induction phenotype (Figure 4 and Table 1).

### 3.4. Effects of CLI on Extracellular NADase Activity in emm89 Strains Having an Inactive CovS Allele

**Figure 5:**
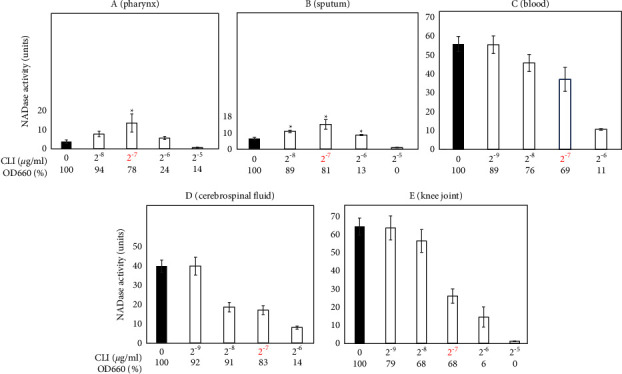
Extracellular NADase activity in five *emm*89 strains isolated from the (A) pharynx, (B) sputum, (C) blood, (D) cerebrospinal fluid, and (E) knee joint of a single patient. Bacteria were cultured with CLI (2^−9^ to 2^−5^ *μ*g/ml). NADase activity was determined based on the criteria described in Figure 1. The OD_660_ of overnight culture with CLI is shown by a percentage (%) of that without CLI. The maximum CLI concentration that did not suppress the bacterial growth to less than 50% of that without CLI is shown in red, which was 2^−7^ *μ*g/ml in all five strains. At least three independent experiments were performed. Error bars indicate the standard errors of the means. Significant increases in extracellular NADase activity by CLI treatment when compared with that without CLI treatment, which is shown by the black bar, are marked with asterisks (^*∗*^*p* < 0.05 using Student's *t*-test).

Recently, we simultaneously isolated five *S. pyogenes* strains from the pharynx, sputum, knee joint, cerebrospinal fluid, and blood of a single STSS patient [18]. All five strains of *S. pyogenes* were derived from a single emm89 clone. The three strains from the knee joint, cerebrospinal fluid, and blood, but not the other two strains, contained a mutation in the *covS* gene to lose its function. As shown in Figure 5, the two strains from the pharynx and sputum showed the CLI-dependent NADase induction phenotype, whereas the other three strains, which had an inactivated *covS* allele, did not.

### 3.5. Effects of CLI on Extracellular NADase Activity in a CLI-Resistant emm1 Strain

**Figure 6:**
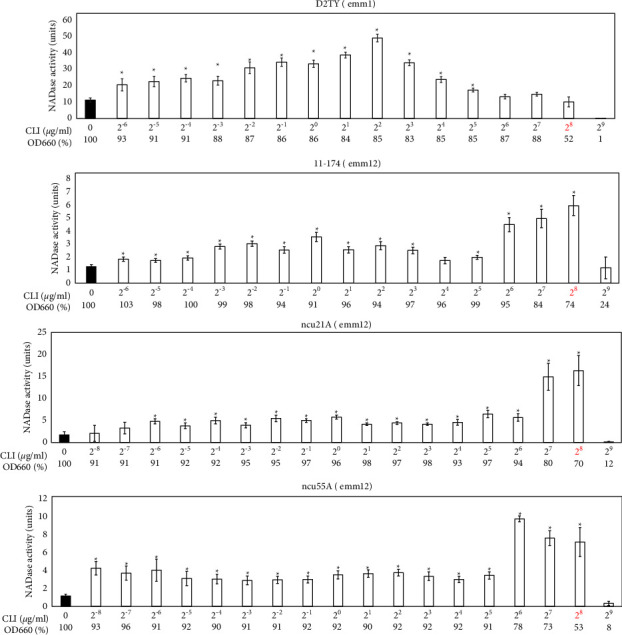
Extracellular NADase activity in the four CLI-resistant *emm*1-or *emm*12-type strains. Bacteria were cultured with CLI (2^−6^ to 2^9^ *μ*g/ml). NADase activity was determined based on the criteria described in Figure 1. The OD_660_ of overnight culture with CLI is shown by a percentage (%) of that without CLI. The maximum CLI concentration that did not suppress the bacterial growth to less than 50% of that without CLI is shown in red, which was 2^8^ *μ*g/ml in all four strains. At least three independent experiments were performed. Error bars indicate the standard errors of the means. Significant increases in extracellular NADase activity by CLI treatment when compared with that without CLI treatment, which is shown by the black bar, are marked with asterisks (^*∗*^*p* < 0.05 using Student's *t*-test).

In *S. pyogenes,* CLI-resistant strains have been identified worldwide. We reported that a CLI-resistant strain, D2TY, induced the expression of NADase by treatment with 1 (=2^0^) *μ*g/ml CLI [17]. However, it is unknown whether the dose (2^0^ *μ*g/ml) of CLI was the best concentration to induce the expression of the NADase because other concentrations were not tested. Therefore, we examined the previous study using various CLI concentrations. As shown in Figure 6, extracellular NADase activities were increased from 2^−6^ to 2^2^ *μ*g/ml of CLI in a dose-dependent manner, and the NADase activity reached the maximum at a concentration of 2^2^ *μ*g/ml CLI, which was not the subinhibitory dose. Furthermore, surprisingly, extracellular NADase activities were decreased from 2^2^ to 2^9^ *μ*g/ml CLI in a dose-dependent manner. In the 2^8^ *μ*g/ml CLI, the subinhibitory dose for this strain, the extracellular NADase activity was equivalent to that without CLI treatment (*p*=0.88). These results indicated that the subinhibitory CLI dose itself is not critical to increase the extracellular NADase activity in at least this D2TY strain.

### 3.6. Effects of CLI on Extracellular NADase Activity in CLI-Resistant emm12 Strain

We further investigated whether the above result was restricted in the *emm*1 D2TY strain. We used three CLI-resistant *emm*12 strains: 11–174, ncu21A, and ncu55A (Figure 6), because D2TY was the only CLI-resistant *emm*1 strain in our collection. The strains 11–174, ncu21A, and ncu55A showed increased extracellular NADase activities after treatment with 2^−6^, 2^−6^, and 2^−8^ *μ*g/ml CLI (*p* < 0.05, <0.01, and <0.01), respectively. These results also indicated that the subinhibitory doses themselves, which were 2^8^ *μ*g/ml CLI in all three strains, were not critical to increase the extracellular NADase activities.

## 4. Discussion

Regarding the CLI-susceptible *S. pyogenes* strains in this study, we updated the information about the CLI-dependent NADase induction phenotypes presented in the previous study [6], in the following regards: (i) we confirmed that the increases in extracellular NADase activity by CLI treatment were statistically significant (Table 1 and Figures 1–5); such statistical analysis was not performed in the previous study [6] because the previous method was not appropriate for statistical analysis (See Introduction section). (ii) We increased the number of strains tested from 5 in the previous study to 23 (Table 1); for example, the number of *emm*1 strains tested was increased from one to five. This enabled us to reveal that the CLI-dependent NADase induction could be a general phenotype in *emm*1-type *S. pyogenes* because all five *emm*1 strains tested showed the CLI-dependent NADase induction (Table 1 and Figure 1). The number of both *emm*4 and *emm*12 strains tested increased from one to three. Because all the strains tested showed the CLI-dependent NADase induction, it may be a general phenotype in *emm*4 and *emm*12 strains as well as the *emm*1 strain (Table 1 and Figure 3). (iii) We analyzed four *emm*89 strains, and all *emm*89 strains showed CLI-dependent NADase induction (Table 1 and Figure 4); *emm*89 strains were not analyzed in the previous study [6] because *emm*89-type strains were not prevalent at that time.

The four strains, SSI-1, 13-O-8, 13-O-10, and 15-T-11, that did not show the CLI-dependent NADase induction phenotype were all *emm*3 genotypes (Table 1 and Figure 2). However, we do not think the *emm*3 genotype itself is a critical factor for the no NADase induction phenotype, because the two *emm*3 strains, ncu6B and ncu62A, showed the NADase induction phenotype (Table 1 and Figure 2). The four strains having the no NADase induction phenotype showed higher NADase activities than the remaining 19 strains (especially, SSI-1 was highest) when they were cultured without CLI (Table 1 and Figure 2). This higher NADase activity observed in the SSI-1 is caused by a *rocA* gene mutation [19,20]. Because RocA represses the production of NADase through its ability to enhance CovR/S system function [21], the phenotype caused by a *rocA* mutation becomes similar to that caused by a *covS* mutation. In fact, the three *emm*89 strains with *covS* mutations showed similar phenotypes to that shown in SSI-1 (Figures 2 and 5).

Regarding the CLI-resistant *S. pyogenes* strains, we also updated the information about the CLI-dependent NADase induction presented in the previous study [17] in the following regards: (i) We confirmed that the increases in extracellular NADase activity by CLI treatment were statistically significant (Figure 6); such statistical analysis was not performed in the previous study because the previous method was not appropriate for statistical analysis (see Introduction section). (ii) We increased the number of strains analyzed from one in the previous study to four, and all four strains showed the CLI-dependent NADase induction phenotype (Figure 6). This enabled us to suggest that the CLI-dependent NADase induction could be a general phenotype in CLI-resistant *S. pyogenes* strains as well as in CLI-susceptible *S. pyogenes* strains. (iii) The subinhibitory doses of CLI themselves did not appear to be critical to increase the extracellular NADase activity. Especially, in strain D2TY, but not in the other three strains (11–174, ncu21A, and ncu55A) the treatment with the subinhibitory dose of CLI did not induce the extracellular NADase activity (Figure 6). This difference between strain D2TY and the other three strains could be related to their *emm*-types, because D2TY has the *emm*1-genotype and the other three strains have the *emm*12-genotype. In order to investigate this question, other *emm*1-type CLI-resistant strains should be investigated.

In this study, 19 of the 23 CLI-susceptible strains tested showed similar NADase induction phenotypes (Table 1 and Figures 1–4). These results indicate that the NADase induced by CLI treatment is not restricted to specific strains and it could be a standard phenotype in *S. pyogenes*. Although the CovRS system is related to the mechanism for NADase induction, as described above (see also Figure 5), we do not believe that the CovS sensor protein has a direct interaction with CLI. For example, CLI may repress the translation of CovS and/or CovR to cause the derepression of *nga*. In our future work, we plan to provide evidence for this hypothesis.

## Figures and Tables

**Table 1:** NADase activity of culture supernatant after administration of CLI in *S. pyogenes* strains.

Strain	*emm* type	NADase activity (U) without CLI^a^	CLI-dependent NADase induction^b^	Subinhibitory dose (*μ*g/ml)	STSS/non-STSS	Reference
(CLI-sensitive)						
1529	1	13.1	+ (3.2)	2^–6^	STSS	[14]
11T-3	1	7.33	+ (5.7)	2^–6^	Non-STSS	[16]
10–85	1	4.51	+ (2.9)	2^–6^	STSS	[14]
11–171	1	6.6	+ (3.1)	2^–6^	STSS	[16]
12–5	1	8.16	+ (2.8)	2^–5^	STSS	[14]
ncu6B	3	3.91	+ (2.4)	2^–6^	Non-STSS	This study
ncu62A	3	34.1	+ (1.4)	2^–6^	STSS	This study
SSI-1	3	82.8	- (N/A)	2^–6^	STSS	[13]
13-O-8	3	58.6	- (N/A)	2^–6^	Non-STSS	This study
13-O-10	3	39.4	- (N/A)	2^–6^	Non-STSS	This study
15T-11	3	49.5	- (N/A)	2^–6^	Non-STSS	This study
ncu33A	4	18.4	+ (2.2)	2^–6^	Non-STSS	This study
ncu115B	4	25.8	+ (1.7)	2^–6^	Non-STSS	This study
ncu117B	4	36.0	+ (1.5)	2^–6^	Non-STSS	This study
NU-syoni	6	16.0	+ (1.7)	2^–7^	STSS	This study
ncu44A	12	3.4	+ (1.9)	2^–6^	Non-STSS	This study
ncu47A	12	4.1	+ (1.6)	2^–6^	Non-STSS	This study
ncu69A	12	4.4	+ (2.6)	2^–6^	Non-STSS	This study
ncu28A	28	7.2	+ (2.6)	2^–6^	STSS	This study
Seibu	89	2.2	+ (3.2)	2^–6^	STSS	This study
ncu52A	89	8.15	+ (1.7)	2^–7^	Non-STSS	This study
ncu60A	89	7.42	+ (2.4)	2^–6^	STSS	This study
ncu61A	89	2.53	+ (3.8)	2^–5^	Non-STSS	This study

(CLI-resistant)						
D2TY	1	12.9	+ (4.6)	2^8^	STSS	[17]
11–174	12	1.30	+ (5.1)	2^8^	STSS	This study
ncu21A	12	1.72	+ (9.4)	2^8^	STSS	This study
ncu55A	12	1.14	+ (8.5)	2^8^	Non-STSS	This study

^a^Basal (in the absence of CLI) levels of NADase activities. Bacteria were cultured without CLI. U: Units, one unit of NADase activity is defined as the amount (*μ*g) of *β*-NAD cleaved per hour per *μ*l culture supernatant, as described previously [10]. ^b^Whether the extracellular NADase activity of each strain was increased with CLI treatment (+) or not (−) was determined based on the experimental results shown in Figure 1 to 6. CLI-dependent NADase activity induction was expressed as values of maximum fold-induction by CLI in parentheses. N/A, not applicant.

## Data Availability

The data that support the findings of this study are available from the corresponding author upon reasonable request.

## Abbreviations

STSS: Streptococcal toxic-shock syndrome
BHI: Brain heart infusion broth
NADase or Nga: NAD-glycohydrolase.
